# Health-Related Quality of Life Among Unaccompanied Refugee Minors Settled in a Norwegian Municipality

**DOI:** 10.1007/s40653-025-00698-x

**Published:** 2025-03-08

**Authors:** Oda Marie Heimli, Ingrid Kvestad, Tormod Bøe, Marit Hjellset Larsen, Nawar Sayyad, Sølve Randal, Kristin Gärtner Askeland

**Affiliations:** 1 RKBU Vest, NORCE Norwegian Research Centre, Bergen, Norway; 2 Department of Psychosocial Science, Faculty of Psychology, University of Bergen, Bergen, Norway; 3 Department of Health Promotion and Development, Faculty of Psychology, University of Bergen, Bergen, Norway; 4 Child Welfare Services for Unaccompanied Refugee Minors, Bergen Municipality, Norway

**Keywords:** Quality of life, HRQoL, Unaccompanied refugee minors, Child welfare, Adolescents, Mental health

## Abstract

The study aimed to examine health-related quality of life (HRQoL) in a group of unaccompanied refugee minors (URMs) and to investigate associations between HRQoL and potential risk and protective factors. The sample includes 79 URMs aged 15 to 20 (83.3% male; 80% response rate) who responded to the KIDSCREEN-27 as a measure of HRQoL. URMs reported lower scores on the HRQoL Index (d = 0.5), *physical well-being* (d = 0.8), *psychological well-being* (d = 0.7), *autonomy and caregiver relations* (d = 0.5), and *peers and social support* (d = 0.5), but not *school environment* (d = 0.04) compared to European population norms. Participants with fewer symptoms of depression had better HRQoL across all dimensions, and URMs with fewer post-traumatic symptoms and less frequent contact with contact persons reported better *psychological well-being*. All effect sizes ranged from small to medium. Contact with family in the home country was not significantly associated with HRQoL. Potential traumatic events were negatively associated with *psychological well-being* and *peers and social support* in post-hoc analyses. To conclude, URMs presented lower HRQoL than European population norms, and HRQoL was related to their reported mental health symptoms.

## Introduction

A refugee is a person who had to flee their country of origin as a result of persecution due to race, religion, nationality, membership in a particular group of society, or political views (United Nations, 1951). Unaccompanied refugee minors (URMs) have fled and resettled in a new country before the age of 18 without a parent or legal guardian (Kirkeberg et al., 2022). While all refugees are exposed to adversities and displacement as a result of war and flight, URMs constitute a particularly vulnerable group as they have to cope with these stressors without the support of a caretaker (Bates et al., 2013). Thus, the challenges faced by URMs extend beyond the initial trauma of displacement (Hodes et al., 2008; Jakobsen et al., 2017; Jensen et al., 2015; Vervliet et al., 2014a) and adjusting to a new culture and environment in their new country can be especially challenging (Makarova et al., 2021). Research from Norway suggests that many URMs suffer from high levels of ongoing war-related intrusive symptoms and depression (Oppedal & Idsoe, 2015), and the mental health problems reported by URMs also seem to persist over time (Jakobsen et al., 2014; Jensen et al., 2019; Keles et al., 2017; Vervliet et al., 2014a).

In Norway, URMs must register at the police station and apply for residency upon arrival. The URMs that arrive at ages 15 to 18 are usually placed at asylum centers for URMs, while URMs under the age of 15 are placed in care centers. All URMs are then assessed before settlement in a Norwegian municipality. The asylum process can be arduous, but the majority of asylum applications from URMs are granted. In 2018, 83% of the applications were granted, and in 2019, 87% were granted (The Norwegian Directorate of Immigration, 2019, 2020). At all stages of the asylum process, the URMs are offered appropriate kindergarten or schooling (The directorate of Integration and Diversity, 2021). The organizational system for URMs varies significantly between municipalities in Norway; however, child welfare services (CWS) are responsible for supporting URMs in most of the larger municipalities and the refugee services in some of the smaller municipalities. These organizational differences can impact the support and services URMs receive, but the municipalities’ economies and regulations are also crucial (Dalgard et al., 2018; Garvik et al., 2016). Settlement in municipalities is conducted regardless of whether the URM is approved for permanent or temporary asylum. When appointed a municipality of settlement, the municipality is responsible for organizing appropriate living arrangements and support for each URM (The directorate of Integration and Diversity, 2021).

While knowledge of the risk and vulnerability inherent in the lives of URMs is crucial to deliver the necessary support services, it is also important to focus on broader health outcomes and the daily life functioning of these adolescents. Several studies suggest that URMs adapt well despite their adverse experiences (Keles et al., 2018; Rodriguez & Dobler, 2021) and it is, therefore, essential to identify factors that can underlie positive development after resettlement. Health-related quality of Life (HRQoL) is a broad, multidimensional concept encompassing physical, psychological, social, and environmental factors that significantly impact a person’s overall well-being (Ravens-Sieberer & Kidscreen Group, 2006; World Health Organization, 2012). HRQoL offers a measure of well-being and function in light of the individual’s goals, expectations, standards, and concerns and in the context of their culture and value system (World Health Organization, 2012). Thus, it is possible to capture culturally sensitive well-being among URMs by incorporating HRQoL measures in both research and practice. Knowledge about the HRQoL among URMs and the factors associated with it could provide a better understanding of potential pathways for support and resilience (Ravens-Sieberer & Kidscreen Group, 2006).

A few studies have investigated HRQoL among adolescent refugees in Scandinavia. When comparing scores to population norms, a study of Syrian adolescent refugees settled in Norway found lower HRQoL on all dimensions except *school environment*, with effect sizes between 0.2 and 0.9 (Dangmann et al., 2020). A study among refugee minors settled in Sweden reported lower HRQoL on dimensions related to *psychological well-being* and *peers and social support*, while the refugees scored higher on *autonomy and parent/guardian relations*, and *school environment* compared to population norms. Notably, a subsample of URMs scored significantly lower on all HRQoL dimensions compared to accompanied adolescents (Solberg et al., 2022). Although differences between the URMs and the reference group were not investigated statistically, the scores for URMs were lower than the European norms for all dimensions except for *school environment*. Taken together, the current knowledge suggests that refugees have lower HRQoL compared to adolescents in the general population, particularly on the *psychological well-being* and *peers and social support* dimensions. Moreover, as findings indicate lower HRQoL for URMs compared to accompanied refugees, there is a need to further examine the HRQoL of URMs in particular.

Studies on the HRQoL of adolescent refugees suggest that their response patterns on the different HRQoL dimensions differ from the response patterns in the population norms. While the norm groups scored highest on *peers and social support*, the refugees scored highest on *autonomy and parent relations* and *school environment* (Dangmann et al., 2020; Solberg et al., 2022). This pattern was also found among a sub-sample of URMs (Solberg et al., 2022).

In general, children with mental health problems have shown compromised quality of life (QoL) in several domains (Dey et al., 2012). Being exposed to adversities during formative years can have a great impact, particularly for URMs who might experience it without the support of parents or guardians (Nilsen et al., 2022). URMs have been found to exhibit more mental distress, symptoms of depression, anxiety, and post-traumatic stress disorder (PTSD) compared to accompanied refugee minors (Daniel-Calveras et al., 2022; Höhne et al., 2022; Kien et al., 2018) and identifying associations between HRQoL and mental health is of importance to develop targeted interventions to improve HRQoL and positive adaptation also in the face of mental health problems (Sharpe et al., 2016). Although few studies have investigated the association between HRQoL and mental health problems particularly among URMs, studies among adolescent refugees have shown that lower QoL was associated with higher levels of depressive symptoms among adolescent refugees from Syria (Erol et al., 2023) and with both depressive symptoms and posttraumatic symptoms (PTS) in a small group of young adult refugees, including URMs (Laufer et al., 2022). QoL was also found to mediate the relationship between resilience and depression, indicating that higher resilience was correlated with improved QoL, which in turn was linked to lower levels of depression (Laufer et al., 2022). Both these studies used a composite score of QoL, and it would be interesting to investigate associations with different dimensions of QoL, as this could inform where interventions to bolster daily life functioning and positive development could be targeted.

Experiencing adverse and traumatic events is considered a risk factor for mental health problems, including PTS, depression, and anxiety among URMs (Baba & Colucci, 2017; Bean et al., 2007; Derluyn et al., 2009; Hodes et al., 2008; Jensen et al., 2015; Smid et al., 2011; Vervliet et al., 2014b). Additionally, the severity of the traumatic events seems to impact the severity of the mental health problems URMs face (Baba & Colucci, 2017). Although limited to refugee samples, adverse experiences both before and during flight have been linked to the HRQoL of adolescent refugees. Experiencing a higher number of potentially traumatic events (PTEs) was associated with lower self-reported HRQoL among adolescent refugees settled in Norway and Australia (Dangmann et al., 2020, 2021; Ziaian et al., 2016). While PTEs were negatively associated with HRQoL in the Norwegian study, the effect was mediated by post-migration stressors and in serial with mental distress. PTEs also influenced HRQoL indirectly through increased risk for PTSD, which led to higher levels of post-migration stress and, in turn, lower HRQoL. Additionally, in contrast to general mental distress, PTSD was not a mediator between PTEs and HRQoL (Dangmann et al., 2021).

Many URMs emphasize the importance of maintaining contact with parents, siblings, and extended family in the home country, seemingly as a source of coping and resilience (Seidel et al., 2022). Indeed, social support from family has been associated with support from mentors and peers in the new country, underlining its importance (Sierau et al., 2018). Several studies have identified an association between social support and mental health problems (Höhne et al., 2022; Oppedal & Idsoe, 2015; Reavell & Fazil, 2017; Sierau et al., 2018), where more support is associated with fewer mental health problems. Further, less mentor support has been associated with an increased risk for depression, anxiety symptoms, and post-traumatic symptoms, underlining the importance of social networks for mental health. A previous study has shown that frequent contact with the family in the home country, but not frequent contact with their contact person in the CWS, was associated with more protective factors in a sample of URMs (Heimli et al., 2024). As HRQoL is a broad concept that covers several aspects of a person’s well-being beyond mental health problems, it would be of interest to examine whether social support from important people in the lives of URMs is associated with their HRQoL across dimensions.

The primary aim of this study was to examine HRQoL as measured by the KIDSCREEN-27 among a group of URMs settled in Norway. Based on the limited previous research, we expected URMs to report lower overall HRQoL compared to European norms. A secondary aim was to examine the relationship between HRQoL and symptoms of depression and post-traumatic stress disorder (PTSD), contact with family in the home country, and contact with the contact person from the CWS. Based on previous research, we expected symptoms of both depression and PTSD to be negatively associated with HRQoL while having frequent contact with family and frequent contact with contact person to be positively associated with HRQoL.

## Method

### Participants

The project was developed in cooperation with the CWS for URMs in Bergen municipality. Participants settled in Bergen municipality were recruited through the CWS, and inclusion criteria were being 15 years or older and in contact with the CWS. A total of 101 URMs were invited to participate, and 81 consented to participate, of which 79 responded to the measure of HRQoL.

### Design and Setting

The present study was part of the larger research project “Pathways to Independence” (PTI) (Kvestad, Randal, et al., 2023), a comprehensive cross-sectional survey conducted in Bergen municipality between December 2018 and January 2019. The survey was developed in close cooperation with the CWS particularly for this project, and included standardized questionnaires on mental health, resilience, and HRQoL and questions on demography, living situation, schooling, contact with the CWS, and social support. The survey was undertaken in the offices of the URMs’ contact person at the CWS. A pilot study among older URMs indicated that Norwegian was suitable for most of the respondents with reformulations and simplifications, and thus the questionnaires were administered in Norwegian. In our sample, six of the URMs needed interpreters. The contact persons from the CWS were also available for questions, support, and follow-up when needed during the survey. Additionally, the URMs in staffed homes were given extra attention and support when needed following the survey. The entire survey took between 1.5 and 3.5 h to fill out.

## Measures

The URMs reported age, gender, and country of origin. Years since arrival were calculated by subtracting the age at participation from the age at arrival. Contact with family was measured with the question: “Do you have family in your home country that you stay in contact with?” with response alternatives “no contact,” “daily,” “weekly,” “monthly,” and “less frequently.” The frequency of contact with family was dichotomized into two groups, where daily contact and weekly contact were collapsed into *contact weekly or more often*, and monthly, less frequently, and no contact into *contact monthly or less frequently.* Contact with the contact person was measured with the question, “How often do you communicate with your contact person in the CWS?” with response alternatives “every day,” “2–4 times a week,” “Once a week,” “2–3 times a month” and “less frequently.” The frequency of contact with the contact person was dichotomized into two groups, where daily or weekly contact was collapsed into *contact weekly or more often* and 2–3 times a month and less frequently into *contact 2–3 times a month or less frequently.*

KIDSCREEN-27 was used to measure HRQoL. KIDSCREEN-27 is cross-culturally validated in 38 languages, including Norwegian (Haraldstad et al., 2006). The questionnaire is generic self-report, and measures behaviors and feelings experienced the last week (Ravens-Sieberer et al., 2014). The measure consists of 27 questions, rated on a 5-point Likert scale with response options from “never” (1) to “always” (5). A total of 4 items were reversed when scoring the questionnaire (Ravens-Sieberer & Kidscreen Group, 2006). The questions in KIDSCREEN-27 are divided into five dimensions: *physical well-being* (5 items), *psychological well-being* (7 items), *autonomy and parent-child relation* (7 items), *peers and social support* (4 items), and *school environment* (4 items) (Ravens-Sieberer & Kidscreen Group, 2006). A general HRQoL index was calculated based on 10 items from KIDSCREEN-27. For the current study, three items in the *autonomy and parent-child relation* dimension regarding parent-child relation were reformulated to focus on adults where they live. This dimension is, therefore, referred to as *autonomy and caregiver relations* in our study. A scoring algorithm was used to calculate T-scores for all the dimensions, with a mean of 50 and a standard deviation of 10. A European consortium developed the scoring algorithm to ease interpretations of scores across samples, with population norm mean T-scores at 50 (Ravens-Sieberer & Kidscreen Group, 2006). The score in each dimension only includes respondents with 0 or 1 missing item. Therefore, all respondents with two or more missing items in a dimension were not included in that specific dimension. A higher score indicates better HRQoL (Ravens-Sieberer & Kidscreen Group, 2006). The KIDSCREEN-27 has shown robust psychometric properties and has been used in refugee samples previously (Dangmann et al., 2020; Ravens-Sieberer et al., 2007; Solberg et al., 2022). All included dimensions had acceptable reliability, indicated by Macdonald’s omegas, with values between 0.79 and 0.89.

Depressive symptoms were measured by the Short Mood and Feelings Questionnaire (SMFQ) (Angold et al., 1995). SMFQ is a questionnaire of 13 questions that are rated on a 3-point Likert scale with response options “not true” (0), “sometimes true” (1), and “true” (2). The questionnaire measures feelings and experiences related to depressive symptoms in the last two weeks. SMFQ is validated for use in the general population (Angold et al., 1995; Turner et al., 2014), but not in refugee/cross-cultural samples. The reliability of SMFQ measured with Macdonald’s omega was 0.87 in this sample.

CATS is a two-part questionnaire where part one measures PTEs and part two measures post-traumatic symptoms (Sachser et al., 2017). CATS part one measured PTEs through 18 items regarding interpersonal violence outside the family, abuse in the family, and sexual abuse. The responses were scored as No (0), Pass (0), or Yes (1). In our project, only the URMs who reported experiencing at least one PTE responded to part two. CATS part two was used to measure post-traumatic symptoms. The questionnaire contains 20 items divided into four factors: intrusions, avoidance, negative alterations in cognition and mood, and hyper-arousal (Sachser et al., 2017). The responses were rated on a 4-point Likert scale with response options from “never” (0) to “almost always” (3). A score above 21 and at least one symptom in each factor indicates probable PTSD, according to recommendations (Sachser et al., 2022). The CATS measure has shown moderate to strong validity in various populations (Dowdy-Hazlett et al., 2021; Sachser et al., 2017, 2022); however, it has not been validated among URMs specifically. The reliability of the CATS part two, measured by Macdonald’s omega, was 0.92.

### Statistical Analyses

Welch t-tests were conducted to compare self-reported HRQoL in a group of URMs to a normative sample. Due to different variances between the two samples, Welch t-tests were considered the best option. The results from the t-tests were used to calculate Cohen’s d effect sizes. These were interpreted according to Cohen (Cohen, 1988) with a small effect being 0.2, a medium effect as 0.5, and a large effect as 0.8. P-values are presented at a 95% significance level.

Bivariate regression analysis investigated associations between HRQoL and symptoms of depression, post-traumatic symptoms, gender, and contact with family in the country of origin. The sum scores for depression and post-traumatic symptoms were standardized with a mean of 0 and a standard deviation (SD) of 1. In post hoc analysis, the association between PTEs and HRQoL was also investigated in bivariate regression analysis.

All analyses were performed using STATA SE version 17.0 or 18.5.

## Results

Table 1 presents the demographic characteristics of the sample. The sample consisted of 17.7% girls, and the most common country of origin was Afghanistan (46.8%). The mean time since arrival was 3.5 years, with 25% of the participants having a time since arrival above the mean. The URMs reported a mean score of 6.8 (SD = 4.9) on symptoms of depression. Additionally, the participants reported a mean total post-traumatic symptom score of 16.0 (SD = 11.9), with 35% reporting scores indicating a probable PTSD diagnosis.

**Table 1:** Sociodemographic characteristics of the participants in the Pathways to Independence study (*N* = 79)

	*n*	%	Mean	SD	Min	max
**Age**			17.96	1.32	15	20
**Female gender**	14	17.72				
**Time since arrival (years)**			3.51	2.25		
Participants above mean	20	25.32				
**Country of origin**						
Afghanistan	37	46.84				
Syria	14	17.72				
Eritrea	13	16.46				
Somalia	7	8.86				
Other^a^	8	10.13				
**SMFQ** ^**b**^	79		6.81	4.87	0	20
**CATS total score** ^**c**^	71		15.96	11.92	0	47
Participants above cut-off (>21)	25	35.21				
**Number of PTEs** ^**d**^	78		6.03	3.59	0^e^	16
Interpersonal violence	74	93.67				
Sexual abuse	15	18.99				
Abuse	40	50.63				
Neglect	42	53.16				
Experienced confusion or helplessness	39	46.37				

^a^Ethiopia, Palestine, Congo, and Sudan

^b^SMFQ = Short Mood and Feelings Questionnaire (symptoms of depression)

^c^PTEs = potentially traumatic events

^d^CATS = post-traumatic symptoms

^e^Three participants reported no PTEs

Our sample reported a lower mean HRQoL Index score than European population norms, with a medium effect size (d = 0.52, see Table 2). The participants reported lower mean scores on the dimensions *physical well-being*, *psychological well-being*, *autonomy and caregiver relations*, and *peers and social support* compared to European population norms, with the differences corresponding to small to medium effect sizes (Range = 0.48–0.76). There was no significant difference in the means between the two groups on the dimension *school environment*.

**Table 2:** T-scores on the KIDSCREEN-27 dimensions in 79 unaccompanied refugee minors and comparison with European population norms

HRQoL dimensions^a^	URMs^b^ (15-20 years)			European population norms (12-18 years)^c^			t	*p*	Cohen’s d
	*n*	M	SD	*n*	M	SD			
Physical well-being	79	42.02	7.38	15,239	48.57	9.64	7.85	<0.001	0.76
Psychological well-being	79	42.38	9.58	15,323	48.83	9.78	5.97	<0.001	0.67
Autonomy and caregiver relations	70	44.47	10.49	15,135	49.41	9.81	3.93	<0.001	0.49
Peers and social support	79	44.21	12.26	15,372	49.62	9.96	3.92	<0.001	0.48
School environment	76	47.98	11.33	15,255	48.44	9.41	0.35	0.725	0.04
HRQoL Index	76	43.74	9.15	14,932	48.51	9.28	4.53	<0.001	0.52

Results from Welch t-tests

aHRQoL = Health-Related Quality of Life

^b^URM = unaccompanied refugee minors

^c^European norm data from the construction and validation of the KIDSCREEN instruments

Including youth from 13 European countries (Ravens-Sieberer & Kidscreen Group, 2006)

Table 3 shows the association between the dimensions of KIDSCREEN-27 and symptoms of depression, post-traumatic symptoms, contact with family in the home country, and contact with the contact person in the CWS. There were significant negative associations between symptoms of depression and all HRQoL dimensions, with coefficients between − 6.08 for *psychological well-being* (95% CI= -7.76, -4.40) and − 2.22 for *physical well-being* (95% CI= -3.82, -0.62). Post-traumatic symptoms were also negatively associated with *psychological well-being*, with a coefficient of -4.28 (95% CI= -6.36, -2.20). However, the associations with *physical well-being*, *autonomy and caregiver relations*, *peers and social support*, and *school environment* were not statistically significant. Frequent contact with family in the home country was not significantly associated with any KIDSCREEN-27 dimensions. Frequent contact with the contact person scored significantly lower on *psychological well-being* (Coefficient= -4,69, 95% CI= -9.16, -0.22), but there were no significant differences on the other dimensions.

**Table 3:** Associations between the dimensions of KIDSCREEN-27 and potential predictors in 79 unaccompanied minors

	Physical well-being		Psychological well-being		Autonomy and caregiver relations		Peers and social support		School environment	
	b	95% CI	b	95% CI	b	95% CI	b	95% CI	b	95% CI
Depression symptoms	**-2.22**	**[-3.82**, **-0.62]**	**-6.08**	**[-7.76**, **-4.40]**	**-4.10**	**[-6.44**, **-1.76]**	**-4.41**	**[-7.01**, **-1.82]**	**-3.84**	**[-6.32**, **-1.37]**
PTSD symptoms^a^	-1.30	[-3.11, 0.51]	**-4.28**	**[-6.36**, **-2.20]**	-1.69	[-4.26 0.88]	-1.40	[-4.26, 1.45]	-0.57	[-3.43, 2.30]
Contact with family^b^	-0.07	[-3.58, 3.44]	0.77	[-3.79, 5.32]	-0.81	[-6.17, 4.56]	3.62	[-2.15, 9.39]	-2.09	[-7.52, 3.33]
Contact with contact person^c^	-1.46	[-4.99, 2.07]	**-4.69**	**[-9.16**, **-0.22]**	-4.44	[-9.59, 0.71]	-1.18	[-7.05, 4.70]	-2.36	[-7.88, 3.16]

Depression symptoms and PTSD symptoms were standardized (*z*-transformed) in the models

^a^PTSD = post-traumatic stress disorder

^b^Contact family in home country = weekly or more often vs. less (ref)

^c^Contact with contact person in child welfare services = weekly or more often vs. less (ref)

Significant associations are marked in boldface

To further understand the results regarding post-traumatic symptoms, we included PTEs to investigate whether its association with HRQoL differed from the association with post-traumatic symptoms, as has been suggested in previous research (Dangmann et al., 2021). Only three of the participants reported no PTEs. Almost 94% of the participants had experienced interpersonal violence outside of family, which also includes war and terror. Approximately 50% of the participants had experienced abuse, neglect, and/or anything that made them feel confused or helpless (See Table 1). The results demonstrated that exposure to more PTEs was associated with lower scores on all dimensions of KIDSCREEN-27. However, the association was only statistically significant for *psychological well-being* (b=-3.91, 95% CI=-5.91, -1.92) and for *peers and social support* (b=-4.55, 95% CI=-7.13, -1.97) (See Table 4).

**Table 4:** Associations between the dimensions of KIDSCREEN-27 and the number of potential traumatic events in 79 unaccompanied minors settled in Norway

	Physical well-being		Psychological well-being		Autonomy and caregiver relations		Peers and social support		School environment	
	b	95% CI	b	95% CI	b	95% CI	b	95% CI	b	95% CI
PTEs^a^	-1.15	[-2.82, 0.53]	**-3.91**	**[-5.91**, **-1.92]**	-2.76	[-5.20, 0.31]	**-4.55**	**[-7.13**, **-1.97]**	-1.78	[-4.39, 0.82]

Significant associations are marked in boldface

^a^PTEs = potentially traumatic events

## Discussion

The URMs in our study reported lower general HRQoL compared to European population norms, in line with a study of adolescent refugees (Dangmann et al., 2020). Our participants reported lower mean scores than the population norms on the dimensions *physical well-being*, *psychological well-being*, *autonomy and caregiver relations*, and *peers and social support*, partly in line with previous studies in adolescent refugees (Dangmann et al., 2020; Solberg et al., 2022). Moreover, URMs in the previous study reported significantly lower scores on all HRQoL dimensions (t-scores between 41.1 and 49.3) compared to accompanied adolescents (t-scores between 46.9 and 55.7) (Solberg et al., 2022), corroborating our findings. Except for *psychological well-being*, the t-scores reported for URMs were slightly higher in the previous study, indicating lower self-reported HRQoL in the current study sample.

Interestingly, the scores on the *school environment* dimension did not differ significantly from the European population norms, which is in line with previous research on adolescent refugees (Dangmann et al., 2020; Solberg et al., 2022). Like our study, Dangmann et al. (2020) did not find a significant difference between adolescent refugees and population norms on school environment, while Solberg et al. (2022) reported significantly higher scores on school environment compared to the norms. Further, school environment was the dimension with the highest score among the URMs in our study, indicating that schools can be important resources for well-being and HRQoL among URMs. Schools can provide a sense of stability in addition to opportunities for social integration and social mobility (Oppedal et al., 2017). A recent review on the risk and resilience factors for education of URM shed further light on how various environmental and social support systems can contribute to educational success and well-being. According to the review, educational resilience depends on a complex mix of factors where factors in the macro and microsystems are highlighted as the most important (Aleghfeli & Hunt, 2022). Particularly, the support provided on a macrosystem by the communities and CWS, as well as supportive living arrangements, supportive friends, and teachers, were identified as influential resilience factors. These are factors outside the adolescents that can be targeted to bolster both education and quality of life in URMs.

Fewer symptoms of depression were associated with higher self-reported HRQoL on all dimensions, in line with previous studies of HRQoL among refugees (Erol et al., 2023; Laufer et al., 2022). This suggests that URMs need support and help to treat symptoms of depression to improve their QoL. Some common causes for symptoms of depression among URMs are adverse experiences, separation from family, lack of social support, adjustment or integration problems, inappropriate living conditions, financial difficulties, and health problems (Fazel et al., 2012; Hodes et al., 2008; Höhne et al., 2022). Thus, to improve URMs’ well-being, it could be beneficial to identify and ameliorate any underlying causes (i.e. ensure appropriate living conditions and financial aid) and secure access to appropriate health services and support.

In contrast, post-traumatic symptoms were only significantly associated with *psychological well-being*, despite 35% of the participants reporting scores above the cut-off for a probable PTSD diagnosis. Strong associations between both symptoms of depression and post-traumatic symptoms and *psychological well-being* are to be expected as these constructs are interconnected. Although, to our knowledge, the association between post-traumatic symptoms and HRQoL has not previously been examined solely among URMs, this is contrary to both our expectations and a small refugee sample, where they found an association between lower QoL and PTS (Laufer et al., 2022). Notably, in a previous study of the same sample, comparing this group of URMs with a group of youth in foster care, the post-traumatic-symptom load and distribution of symptoms differed (Kvestad et al., 2023). While the symptoms of youth in foster care were distributed to a larger extent in either high or low scores, and a greater proportion scored above the cut-off, there was a higher sub-clinical symptom load among URMs. This points to some interesting differences between these high-risk groups, where fewer URMs score above the cut-off despite reporting a greater number of PTEs compared to the foster youth (Kvestad et al., 2023). It is, therefore, possible that the lack of association between post-traumatic symptoms and HRQoL in the present study could be due to how URMs experience their post-traumatic symptoms. To further entangle this and gain a better understanding of our results, we examined the association between HRQoL and PTEs as a possible proxy for their traumatic experiences in post hoc analyses. Findings show that the total number of PTEs was significantly associated with the dimensions *psychological well-being* and *peers and social support*, partly in line with previous studies among refugees (Dangmann et al., 2020, 2021; Ziaian et al., 2016) and similar to our results for posttraumatic symptoms. Thus, neither post-traumatic symptoms nor the number of PTEs were associated with HRQoL as consistently as expected in the current study sample. Seen in the context of the consistent results regarding symptoms of depression, it is possible that the adverse experiences and posttraumatic symptoms are handled differently by the URMs than symptoms of depression. It could further be due to the small sample size and limited statistical power to detect significant differences, as the coefficients for all the HRQoL dimensions are in the expected direction, albeit not statistically significant.

In contrast to our hypothesis, there was no association between the frequency of contact with family in the home country and HRQoL. The separation from parents or caregivers can be a risk factor for the URMs’ well-being, both because the separation itself can be traumatizing and because the separation puts the URMs at risk of experiencing other traumatic events (Derluyn & Broekaert, 2007). Being exposed to trauma can create complex needs for URMs, which, for some, can indicate a need for familial support to improve well-being (Fazel et al., 2012). However, mental health research in the general population states that the quality of social interaction impacts well-being and psychological adjustment to a greater extent than the frequency of contact (Baumeister & Leary, 1995; Hartup & Stevens, 1999). This could explain the lack of association, as the frequency of contact could be less important for HRQoL and well-being. Frequent contact with the contact person from CWS was, to our surprise, negatively associated with HRQoL, despite previous studies highlighting the importance of competent and secure adults outside the family to help cope with life stressors for URMs (Derluyn & Broekaert, 2007; Hopkins & Hill, 2008). Although initially surprising, our finding could indicate that URMs with more frequent contact with their contact person have lower psychological well-being and, therefore, need more support and help in their daily life from the CWS compared to URMs with less frequent contact. In other words, our findings can imply that the CWS tailors the support provided to the individual needs of the URMs. Thus, in contrast to previous research stating that more social support is associated with fewer mental health problems among URMs (Höhne et al., 2022; Oppedal & Idsoe, 2015; Reavell & Fazil, 2017; Sierau et al., 2018), the included measures for social support in our study did not seem as evident for their HRQoL. This could, again, be related to the investigation of frequence of contact, and not quality.

### Strengths and Limitations

Our sample is a hard-to-reach group, which is a major strength. Moreover, the study’s high response rate and representativeness in terms of gender distribution and country of origin are strengths. Another strength of this study is the use of a well-validated HRQoL measure that has not been used among a group of URMs previously.

As this is a descriptive cross-sectional study, it is not possible to make causal inferences, and there might be influential factors that we are unable to control for, such as change in HRQoL over time which future studies should try to include in their assessment procedures. Additionally, the sample is settled in one Norwegian municipality, which can limit the generalizability of the results. The larger municipalities in Norway tend to have better economies, and a higher percentage of URMs receive measures compared to the smaller municipalities (Dalgard et al., 2018). Being settled in the same municipality indicates that the URMs in our sample have the same municipality’s economy, organization, and geographic environment.

The comparison between the URMs and the European population norms on the *autonomy and caregiver relations* dimension could be influenced by the reformulated items in our study. Further, there was a higher number of missing on the *autonomy and caregiver relations* dimension (9 respondents missing). This could be due to the specific questions, as the items with missing in the *autonomy and caregiver relations* dimensions were all regarding an adult where they live and some of the URMs live in housing without a responsible adult. The low number of missing items in total, however, indicates that the KIDSCREEN-27 was a well-accepted measure among the URMs.

All measures have been used in refugee populations previously but not validated specifically, which represents a limitation to our study. A complicating factor in the current study is the unintended omission of 1 item in the CATS measure regarding difficulties with sleep, which further complicates the comparison with other studies.

The presence of child welfare workers at the data collection location could have impacted the responses of the participants. There is also a possibility of misunderstanding when filling out the survey. The study took precautions by adapting the language after the pilot study, having interpreters available when needed, and having contact persons available for questions. The Norwegian language may still have been difficult to understand as a second language for some respondents, however. On the other hand, clarifications, trust, support, and a safe environment could have been crucial for the participant to give honest and valid responses.

Although our sample is representative of URMs in Norway in terms of origin, age, and gender (Eurostat, 2015), the sample is relatively small, resulting in limited statistical power to discover smaller differences. Therefore, the uncertainty around some of our estimates is high, suggesting these should be interpreted cautiously.

Because the age composition in our sample and the normative sample differs slightly, we examined the importance of age in our sample. We found a weak positive correlation between age and the dimensions *psychological well-being* and *school environmen*t, while correlations between age and *physical well-being*, *autonomy and caregiver relations*, and *peers and social support* were weak and negative. These weak correlations indicate no large age differences in levels of HRQoL in our sample.

## Conclusion and Implications

URMs in our sample reported lower HRQoL compared to European population norms on most domains, especially on *physical* and *psychological well-being*. The relatively low scores might indicate a need for support and measures targeting improved well-being and HRQoL in several domains of their lives. Notably, the URMs scored within average on the *school environment* domain, implying that they experience their school environment as positive and supportive for their well-being. Thus, our findings highlight the importance of facilitating inclusive and supporting school environments that function as arenas for both socialization and learning for all URMs. This is also important information for the contact persons in CWS, underlining the importance of motivating URMs to attend school. Further, the negative association between symptoms of depression and all dimensions of HRQoL suggests that adapted care and targeted treatment among URMs suffering from symptoms of depression could be an important measure to improve their overall well-being. Surprisingly, post-traumatic symptoms and PTEs were not associated with HRQoL as consistently as expected despite a high burden of PTEs reported in the sample. Symptoms of depression appears to have a greater impact on HRQoL than post-traumatic symptoms, underscoring the importance of identifying specific mental health problems among URMs. In conclusion, our findings suggest a need for increased focus on mental health problems, such as symptoms of depression, and how these influence the everyday well-being of URMs.

## Data Availability

Access to Data is restricted by Norwegian law on medical and health-related research. Information about the data and analysis is available from the corresponding author Oda Marie Heimli on request.
